# (*Z*)-3-Benzyl-1,5-benzothia­zepin-4(5*H*)-one

**DOI:** 10.1107/S1600536812029054

**Published:** 2012-07-04

**Authors:** V. Sabari, R. Selvakumar, M. Bakthadoss, S. Aravindhan

**Affiliations:** a Department of Physics, Presidency College (Autonomous), Chennai 600 005, India; b Department of Organic Chemistry, University of Madras, Chennai 600 025, India

## Abstract

In the crystal structure of the title compound, C_16_H_13_NOS, mol­ecules are linked into cyclic centrosymmetric *R*_2_^2^(8) dimers *via* pairs of N—H⋯O hydrogen bonds. The seven-membered ring adopts a boat conformation.

## Related literature

For the pharmaceutical properties of thia­zepin derivatives, see: Tomascovic *et al.* (2000 ▶); Rajsner *et al.* (1971 ▶); Metys *et al.* (1965 ▶). For conformations of thia­zepin derivatives, see: Huang *et al.* (2011 ▶). For a related structure, see: Sabari *et al.* (2012 ▶).For graph-set analysis of hydrogen bonds, see: Bernstein *et al.* (1995 ▶).
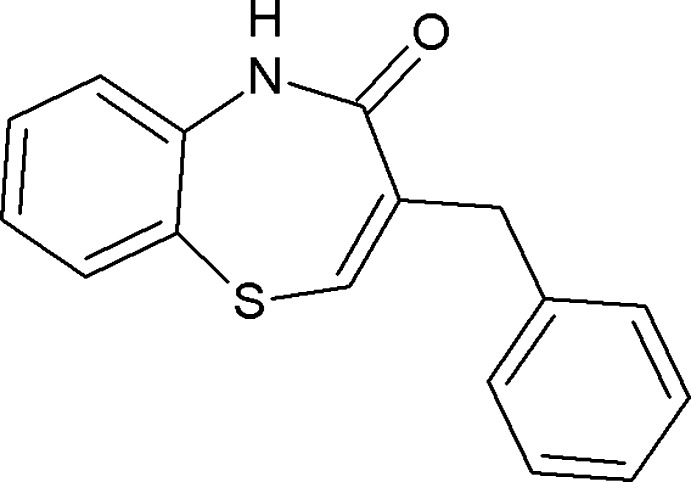


## Experimental

### 

#### Crystal data

C_16_H_13_NOS
*M* *_r_* = 267.33
Monoclinic, 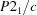

*a* = 9.3409 (6) Å
*b* = 11.7876 (7) Å
*c* = 11.8310 (6) Å
β = 94.727 (3)°
*V* = 1298.24 (13) Å^3^
*Z* = 4
Mo *K*α radiation
μ = 0.24 mm^−1^
*T* = 293 K
0.32 × 0.20 × 0.10 mm

#### Data collection

Bruker SMART APEXII CCD diffractometer
Absorption correction: multi-scan (*SADABS*; Bruker, 2008 ▶) *T*_min_ = 0.980, *T*_max_ = 0.990
11696 measured reflections
3220 independent reflections
2700 reflections with *I* > 2σ(*I*)
*R*_int_ = 0.024

#### Refinement

*R*[*F* ^2^ > 2σ(*F* ^2^)] = 0.036
*wR*(*F* ^2^) = 0.100
*S* = 1.03
3220 reflections
224 parameters
All H-atom parameters refined
Δρ_max_ = 0.25 e Å^−3^
Δρ_min_ = −0.31 e Å^−3^

### 

Data collection: *APEX2* (Bruker, 2008 ▶); cell refinement: *SAINT* (Bruker, 2008 ▶); data reduction: *SAINT*; program(s) used to solve structure: *SHELXS97* (Sheldrick, 2008 ▶); program(s) used to refine structure: *SHELXL97* (Sheldrick, 2008 ▶); molecular graphics: *ORTEP-3* (Farrugia, 1997 ▶); software used to prepare material for publication: *SHELXL97* and *PLATON* (Spek, 2009 ▶).

## Supplementary Material

Crystal structure: contains datablock(s) I, global. DOI: 10.1107/S1600536812029054/bt5942sup1.cif

Structure factors: contains datablock(s) I. DOI: 10.1107/S1600536812029054/bt5942Isup2.hkl

Supplementary material file. DOI: 10.1107/S1600536812029054/bt5942Isup3.cml

Additional supplementary materials:  crystallographic information; 3D view; checkCIF report

## Figures and Tables

**Table 1:** Hydrogen-bond geometry (Å, °)

*D*—H⋯*A*	*D*—H	H⋯*A*	*D*⋯*A*	*D*—H⋯*A*
N1—H⋯O1^i^	0.847 (18)	2.098 (19)	2.9407 (15)	173.1 (16)

Symmetry code: (i) 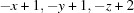.

**Fig. 1.**
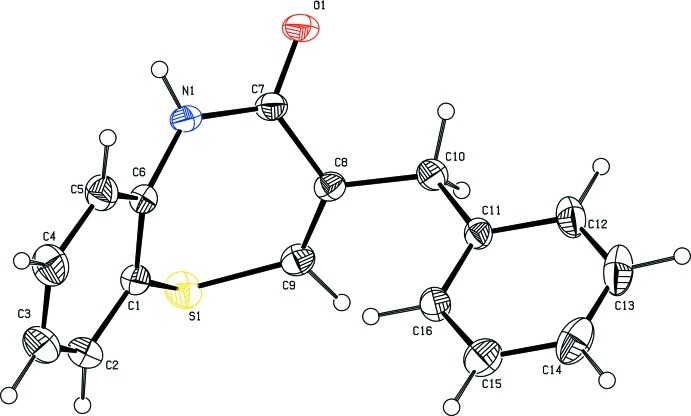
The molecular structure of the title compound, showing 30% probability displacement ellipsoids.

**Fig. 2.**
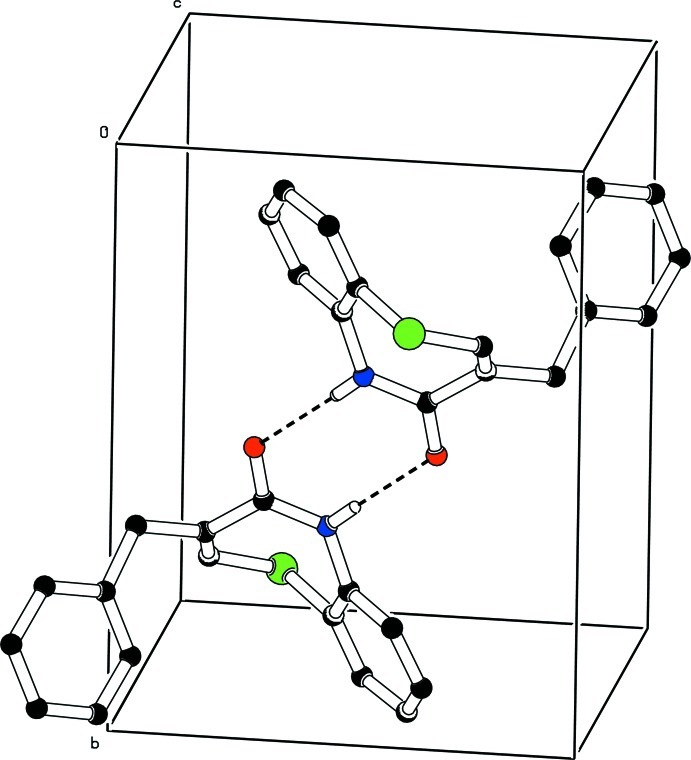
A view of the crystal packing. H atoms not involved in hydrogen bonding (dashed lines) have been omitted for clarity.

## supplementary crystallographic information

### Comment

The title compound is used as an intermediate for the synthesis of dosulepin, which is an antidepressant of the tricyclic family. Dosulepin prevents reabsorbing of serotonin and noradrenaline in the brain, helps to prolong the mood lightening effect of any released noradrenaline and serotonin, thus relieving depression. The dibenzo[c,e]thiazepin derivatives exhibit chiroptical properties (Tomascovic *et al.*, 2000). Dibenzo[b,e]thiazepin-5,5-dioxide derivatives possess antihistaminic and antiallergenic activities (Rajsner *et al.*, 1971). Benzene thiazepin derivatives are identified as a new type of effective antihistaminic compounds (Metys *et al.*, 1965). Considering the wide range of biological activities of the thiazepin derivatives, we determined the crystal structure of the title compound.

X-Ray analysis confirms the molecular structure and atom connectivity as illustrated in Fig. 1. The seven membered thiazepin ring adopts a boat conformation (Huang *et al.*, 2011). The atom O1 deviates by 1.0431 (11) Å from the least-squares plane of the thiazepin ring. The sum of the bond angles around the N1 atom (357.73°) of the thiazepin ring is in agreement with *sp*^2^ hybridization. The molecules are linked into cyclic centrosymmetric dimers *via* N—H···O hydrogen bonds with the motif *R*_2_^2^(8) (Bernstein *et al.*, 1995).

### Experimental

A mixture of (*Z*)-methyl 2-(bromomethyl)-3-phenylacrylate (2 mmol) and *o*-aminothiophenol (2 mmol) in the presence of potassium *tert*-butoxide (4.8 mmol) in dry THF (10 ml) was stirred at room temperature for 1 h. After the completion of the reaction as indicated by TLC, the reaction mixture was concentrated and the resulting crude mass was diluted with water (20 ml) and extracted with ethyl acetate (3 *x* 20 ml). The organic layer was washed with brine (2 *x* 20 ml) and dried over anhydrous sodium sulfate. The organic layer was concentrated, which successfully provide the crude final product ((*Z*)-3-benzylbenzo[*b*][1,4]thiazepin-4(5*H*)-one). The final product was purified by column chromatography on silica gel to afford the title compound 41% yields.

### Refinement

H atoms were freely refined.

### Crystal data

**Table:**

C_16_H_13_NOS	*F*(000) = 560
*M**_r_* = 267.33	*D*_x_ = 1.368 Mg m^−^^3^
Monoclinic, *P*2_1_/*c*	Mo *K*α radiation, λ = 0.71073 Å
Hall symbol: -P 2ybc	Cell parameters from 8725 reflections
*a* = 9.3409 (6) Å	θ = 2.8–29.1°
*b* = 11.7876 (7) Å	µ = 0.24 mm^−^^1^
*c* = 11.8310 (6) Å	*T* = 293 K
β = 94.727 (3)°	Monoclinic, colourless
*V* = 1298.24 (13) Å^3^	0.32 × 0.20 × 0.10 mm
*Z* = 4	

### Data collection

**Table:**

Bruker SMART APEXII CCD diffractometer	3220 independent reflections
Radiation source: fine-focus sealed tube	2700 reflections with *I* > 2σ(*I*)
Graphite monochromator	*R*_int_ = 0.024
Detector resolution: 15.9948 pixels mm^-1^	θ_max_ = 28.3°, θ_min_ = 2.2°
ω and φ scans	*h* = −12→12
Absorption correction: multi-scan (*SADABS*; Bruker, 2008)	*k* = −12→15
*T*_min_ = 0.980, *T*_max_ = 0.990	*l* = −15→15
11696 measured reflections	

### Refinement

**Table:**

Refinement on *F*^2^	Primary atom site location: structure-invariant direct methods
Least-squares matrix: full	Secondary atom site location: difference Fourier map
*R*[*F*^2^ > 2σ(*F*^2^)] = 0.036	Hydrogen site location: inferred from neighbouring sites
*wR*(*F*^2^) = 0.100	All H-atom parameters refined
*S* = 1.03	*w* = 1/[σ^2^(*F*_o_^2^) + (0.0473*P*)^2^ + 0.3853*P*] where *P* = (*F*_o_^2^ + 2*F*_c_^2^)/3
3220 reflections	(Δ/σ)_max_ = 0.001
224 parameters	Δρ_max_ = 0.25 e Å^−^^3^
0 restraints	Δρ_min_ = −0.31 e Å^−^^3^

### Special details

**Table:**

Geometry. All e.s.d.'s (except the e.s.d. in the dihedral angle between two l.s. planes) are estimated using the full covariance matrix. The cell e.s.d.'s are taken into account individually in the estimation of e.s.d.'s in distances, angles and torsion angles; correlations between e.s.d.'s in cell parameters are only used when they are defined by crystal symmetry. An approximate (isotropic) treatment of cell e.s.d.'s is used for estimating e.s.d.'s involving l.s. planes.
Refinement. Refinement of *F*^2^ against ALL reflections. The weighted *R*-factor *wR* and goodness of fit *S* are based on *F*^2^, conventional *R*-factors *R* are based on *F*, with *F* set to zero for negative *F*^2^. The threshold expression of *F*^2^ > σ(*F*^2^) is used only for calculating *R*-factors(gt) *etc*. and is not relevant to the choice of reflections for refinement. *R*-factors based on *F*^2^ are statistically about twice as large as those based on *F*, and *R*- factors based on ALL data will be even larger.

### Fractional atomic coordinates and isotropic or equivalent isotropic displacement parameters (Å^2^)

**Table:**

	*x*	*y*	*z*	*U*_iso_*/*U*_eq_	
H10A	0.9319 (19)	0.4199 (16)	1.0576 (15)	0.054 (5)*	
H14	1.158 (2)	0.0216 (19)	1.2706 (18)	0.071 (6)*	
H13	1.253 (3)	0.210 (2)	1.287 (2)	0.090 (7)*	
H2	0.520 (2)	0.0349 (17)	0.7510 (18)	0.067 (6)*	
H4	0.264 (2)	0.0652 (19)	1.0082 (18)	0.075 (6)*	
H3	0.341 (2)	−0.0401 (19)	0.8584 (18)	0.078 (6)*	
H5	0.367 (2)	0.2445 (15)	1.0477 (16)	0.051 (5)*	
H9	0.906 (2)	0.2452 (13)	0.8225 (15)	0.047 (4)*	
H12	1.152 (2)	0.3537 (18)	1.1720 (16)	0.066 (6)*	
H16	0.8605 (18)	0.1324 (14)	1.0263 (14)	0.048 (4)*	
H15	0.960 (2)	−0.0149 (19)	1.1441 (18)	0.075 (6)*	
H	0.4802 (19)	0.4003 (15)	0.9656 (14)	0.050 (5)*	
H10B	1.0195 (19)	0.3709 (15)	0.9641 (15)	0.053 (5)*	
S1	0.67169 (4)	0.23482 (4)	0.74334 (3)	0.04629 (13)	
O1	0.69525 (11)	0.49768 (9)	1.00015 (11)	0.0515 (3)	
N1	0.54541 (12)	0.35755 (9)	0.94400 (10)	0.0376 (3)	
C9	0.81284 (15)	0.27217 (12)	0.84256 (12)	0.0377 (3)	
C1	0.54547 (14)	0.18101 (11)	0.83286 (11)	0.0361 (3)	
C8	0.80771 (13)	0.33382 (10)	0.93653 (11)	0.0326 (3)	
C7	0.67881 (14)	0.40033 (11)	0.96316 (11)	0.0345 (3)	
C10	0.94322 (15)	0.35387 (12)	1.01201 (13)	0.0397 (3)	
C6	0.49997 (14)	0.24447 (10)	0.92206 (11)	0.0332 (3)	
C11	0.99742 (13)	0.25642 (11)	1.08739 (11)	0.0335 (3)	
C16	0.94145 (15)	0.14763 (12)	1.08123 (12)	0.0396 (3)	
C12	1.11413 (16)	0.27689 (16)	1.16571 (13)	0.0490 (4)	
C5	0.39623 (16)	0.20069 (14)	0.98728 (14)	0.0448 (3)	
C14	1.11727 (19)	0.08314 (19)	1.22626 (15)	0.0599 (5)	
C13	1.17296 (18)	0.1913 (2)	1.23380 (15)	0.0628 (5)	
C2	0.48567 (18)	0.07488 (14)	0.80956 (15)	0.0511 (4)	
C15	1.00050 (19)	0.06142 (15)	1.15013 (14)	0.0508 (4)	
C4	0.33594 (18)	0.09600 (16)	0.96179 (17)	0.0568 (4)	
C3	0.37969 (19)	0.03362 (15)	0.87283 (18)	0.0613 (5)	

### Atomic displacement parameters (Å^2^)

**Table:**

	*U*^11^	*U*^22^	*U*^33^	*U*^12^	*U*^13^	*U*^23^
S1	0.0532 (2)	0.0562 (2)	0.02983 (18)	−0.00076 (17)	0.00540 (15)	−0.00396 (14)
O1	0.0427 (6)	0.0312 (5)	0.0809 (8)	0.0002 (4)	0.0071 (5)	−0.0154 (5)
N1	0.0322 (6)	0.0297 (5)	0.0512 (7)	0.0043 (4)	0.0054 (5)	−0.0075 (5)
C9	0.0372 (7)	0.0390 (7)	0.0381 (7)	0.0021 (5)	0.0096 (5)	0.0012 (5)
C1	0.0350 (6)	0.0356 (7)	0.0370 (6)	0.0033 (5)	−0.0019 (5)	−0.0029 (5)
C8	0.0327 (6)	0.0275 (6)	0.0382 (6)	−0.0004 (5)	0.0067 (5)	0.0038 (5)
C7	0.0363 (6)	0.0280 (6)	0.0396 (6)	0.0018 (5)	0.0054 (5)	−0.0013 (5)
C10	0.0339 (7)	0.0322 (7)	0.0530 (8)	−0.0054 (5)	0.0037 (6)	−0.0020 (6)
C6	0.0301 (6)	0.0311 (6)	0.0377 (6)	0.0021 (5)	−0.0018 (5)	−0.0018 (5)
C11	0.0258 (6)	0.0411 (7)	0.0340 (6)	−0.0021 (5)	0.0057 (5)	−0.0062 (5)
C16	0.0374 (7)	0.0402 (7)	0.0403 (7)	−0.0027 (6)	−0.0011 (5)	0.0001 (6)
C12	0.0338 (7)	0.0670 (10)	0.0457 (8)	−0.0105 (7)	0.0010 (6)	−0.0089 (7)
C5	0.0367 (7)	0.0492 (8)	0.0489 (8)	−0.0044 (6)	0.0063 (6)	−0.0045 (7)
C14	0.0478 (9)	0.0839 (13)	0.0477 (9)	0.0169 (9)	0.0033 (7)	0.0187 (9)
C13	0.0374 (8)	0.1029 (16)	0.0461 (9)	−0.0014 (9)	−0.0081 (7)	0.0013 (9)
C2	0.0507 (9)	0.0421 (8)	0.0593 (9)	0.0009 (7)	−0.0025 (7)	−0.0175 (7)
C15	0.0534 (9)	0.0482 (9)	0.0509 (8)	0.0051 (7)	0.0045 (7)	0.0096 (7)
C4	0.0458 (9)	0.0543 (10)	0.0705 (11)	−0.0141 (7)	0.0066 (8)	0.0015 (8)
C3	0.0529 (10)	0.0412 (9)	0.0888 (13)	−0.0142 (7)	−0.0001 (9)	−0.0104 (9)

### Geometric parameters (Å, º)

**Table:**

S1—C9	1.7479 (15)	C11—C12	1.392 (2)
S1—C1	1.7663 (14)	C16—C15	1.388 (2)
O1—C7	1.2332 (16)	C16—H16	0.972 (17)
N1—C7	1.3460 (17)	C12—C13	1.377 (3)
N1—C6	1.4165 (16)	C12—H12	0.97 (2)
N1—H	0.846 (18)	C5—C4	1.379 (2)
C9—C8	1.3324 (19)	C5—H5	0.942 (19)
C9—H9	0.976 (18)	C14—C13	1.377 (3)
C1—C2	1.388 (2)	C14—C15	1.380 (3)
C1—C6	1.3885 (18)	C14—H14	0.96 (2)
C8—C7	1.4920 (17)	C13—H13	0.96 (3)
C8—C10	1.5064 (19)	C2—C3	1.378 (3)
C10—C11	1.515 (2)	C2—H2	0.92 (2)
C10—H10A	0.958 (19)	C15—H15	0.98 (2)
C10—H10B	0.968 (18)	C4—C3	1.373 (3)
C6—C5	1.387 (2)	C4—H4	0.97 (2)
C11—C16	1.3845 (19)	C3—H3	0.95 (2)
			
C9—S1—C1	101.05 (6)	C11—C16—C15	121.13 (14)
C7—N1—C6	129.99 (11)	C11—C16—H16	118.4 (10)
C7—N1—H	114.0 (12)	C15—C16—H16	120.4 (10)
C6—N1—H	113.8 (12)	C13—C12—C11	121.01 (17)
C8—C9—S1	128.37 (11)	C13—C12—H12	120.7 (12)
C8—C9—H9	118.4 (10)	C11—C12—H12	118.2 (12)
S1—C9—H9	113.2 (10)	C4—C5—C6	120.17 (15)
C2—C1—C6	119.54 (13)	C4—C5—H5	121.1 (11)
C2—C1—S1	118.81 (11)	C6—C5—H5	118.7 (11)
C6—C1—S1	121.57 (10)	C13—C14—C15	119.21 (16)
C9—C8—C7	123.22 (12)	C13—C14—H14	122.2 (13)
C9—C8—C10	119.61 (12)	C15—C14—H14	118.5 (13)
C7—C8—C10	116.46 (12)	C14—C13—C12	120.67 (16)
O1—C7—N1	119.66 (12)	C14—C13—H13	121.2 (15)
O1—C7—C8	119.00 (12)	C12—C13—H13	118.1 (15)
N1—C7—C8	121.30 (11)	C3—C2—C1	120.36 (15)
C8—C10—C11	116.97 (11)	C3—C2—H2	122.7 (13)
C8—C10—H10A	109.6 (11)	C1—C2—H2	117.0 (13)
C11—C10—H10A	109.4 (11)	C14—C15—C16	120.12 (17)
C8—C10—H10B	107.9 (10)	C14—C15—H15	120.0 (12)
C11—C10—H10B	106.0 (11)	C16—C15—H15	119.9 (13)
H10A—C10—H10B	106.3 (15)	C3—C4—C5	120.30 (16)
C5—C6—C1	119.61 (13)	C3—C4—H4	119.7 (13)
C5—C6—N1	117.44 (12)	C5—C4—H4	120.0 (13)
C1—C6—N1	122.71 (12)	C4—C3—C2	119.98 (16)
C16—C11—C12	117.84 (14)	C4—C3—H3	119.7 (13)
C16—C11—C10	124.38 (12)	C2—C3—H3	120.2 (13)
C12—C11—C10	117.75 (13)		
			
C1—S1—C9—C8	−49.95 (14)	C7—N1—C6—C1	−56.6 (2)
C9—S1—C1—C2	−128.63 (12)	C8—C10—C11—C16	8.3 (2)
C9—S1—C1—C6	54.52 (12)	C8—C10—C11—C12	−173.77 (12)
S1—C9—C8—C7	−11.3 (2)	C12—C11—C16—C15	−0.6 (2)
S1—C9—C8—C10	178.70 (10)	C10—C11—C16—C15	177.38 (14)
C6—N1—C7—O1	−167.26 (14)	C16—C11—C12—C13	0.8 (2)
C6—N1—C7—C8	15.0 (2)	C10—C11—C12—C13	−177.31 (14)
C9—C8—C7—O1	−135.97 (15)	C1—C6—C5—C4	−1.7 (2)
C10—C8—C7—O1	34.35 (18)	N1—C6—C5—C4	172.74 (14)
C9—C8—C7—N1	41.8 (2)	C15—C14—C13—C12	−0.6 (3)
C10—C8—C7—N1	−147.91 (13)	C11—C12—C13—C14	−0.2 (3)
C9—C8—C10—C11	−76.59 (17)	C6—C1—C2—C3	1.4 (2)
C7—C8—C10—C11	112.72 (14)	S1—C1—C2—C3	−175.52 (14)
C2—C1—C6—C5	0.5 (2)	C13—C14—C15—C16	0.8 (3)
S1—C1—C6—C5	177.32 (11)	C11—C16—C15—C14	−0.2 (2)
C2—C1—C6—N1	−173.63 (13)	C6—C5—C4—C3	1.0 (3)
S1—C1—C6—N1	3.20 (18)	C5—C4—C3—C2	0.9 (3)
C7—N1—C6—C5	129.13 (16)	C1—C2—C3—C4	−2.1 (3)

### Hydrogen-bond geometry (Å, º)

**Table:**

*D*—H···*A*	*D*—H	H···*A*	*D*···*A*	*D*—H···*A*
N1—H···O1^i^	0.847 (18)	2.098 (19)	2.9407 (15)	173.1 (16)

Symmetry code: (i) −*x*+1, −*y*+1, −*z*+2.
